# Supplementary material for: Prospective and detailed behavioral phenotyping in DDX3X syndrome
Source: Mol Autism. 2021 May 16;12:36. doi: 10.1186/s13229-021-00431-z (PMC8127248; doi:10.1186/s13229-021-00431-z)
Supplement: Supplementary file 2 — Additional file 2: Figure S1.Behavioral comorbidities. A. Frequency histograms for the Child Behavior Checklist (CBCL) Internalizing and Externalizing composite scales, Depressive and Anxiety scales, and Attention-Deficit/Hyperactivity and Defiant DSM-oriented scales for ADHD and oppositional defiant disorder. T-scores have a mean of 50 and standard deviation of 10. B. Frequency histograms for the Vineland-3 Internalizing and Externalizing scales. V scores have a mean of 15 and standard deviation of 3. In all plots, higher scores indicate greater deficits. In panels A & B, distribution of standard scores in typically developing individuals are shown as black lines, together with associated standard deviations (dashed lines). PTV, protein-truncating variant; missense, missense variant or in-frame deletion. [file 13229_2021_431_MOESM2_ESM.pdf]

S1: Behavioral Comorbidities

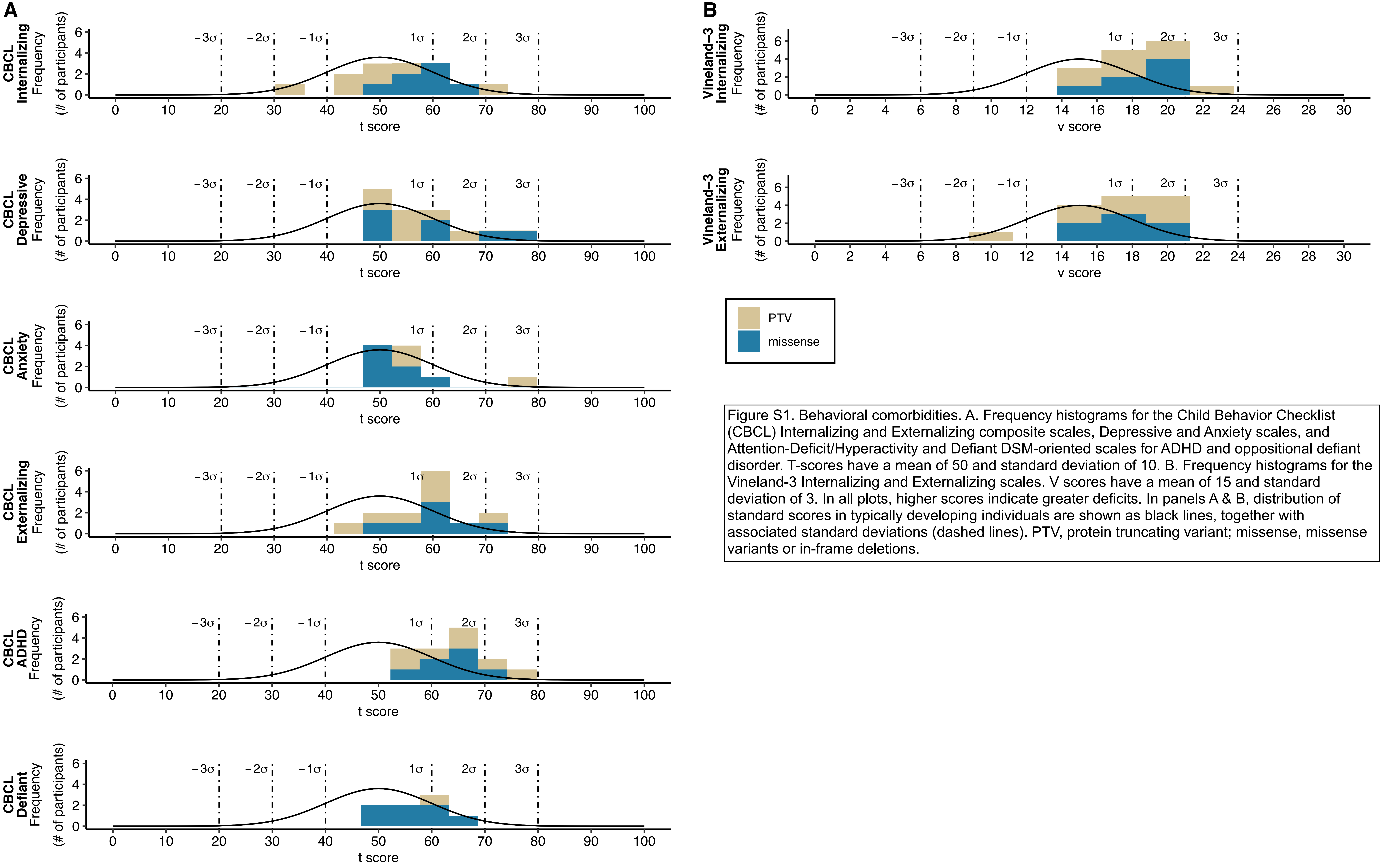

Figure S1. Behavioral comorbidities. A. Frequency histograms for the Child Behavior Checklist (CBCL) Internalizing and Externalizing composite scales, Depressive and Anxiety scales, and Attention-Deficit/Hyperactivity and Defiant DSM-oriented scales for ADHD and oppositional defiant disorder. T-scores have a mean of 50 and standard deviation of 10. B. Frequency histograms for the Vineland-3 Internalizing and Externalizing scales. V scores have a mean of 15 and standard deviation of 3. In all plots, higher scores indicate greater deficits. In panels A & B, distribution of standard scores in typically developing individuals are shown as black lines, together with associated standard deviations (dashed lines). PTV, protein truncating variant; missense, missense variants or in-frame deletions.
